# Evaluation of the Availability and Antioxidant Capacity of Maillard Compounds Present in Bread Crust: Studies in Caco-2 Cells

**DOI:** 10.3390/foods6010005

**Published:** 2017-01-11

**Authors:** Silvia Pastoriza de la Cueva, Isabel Seiquer, Marta Mesías, José Ángel Rufián-Henares, Cristina Delgado-Andrade

**Affiliations:** 1 Departamento de Nutrición y Bromatología, Facultad de Farmacia, Universidad de Granada, Campus Universitario de Cartuja, 18071 Granada, Spain; spdelacueva@ugr.es; 2 Department of Physiology and Biochemistry of Animal Nutrition (EEZ-CSIC), Camino del Jueves, 18100 Granada, Spain; isabel.seiquer@eez.csic.es; 3 Institute of Food Science, Technology and Nutrition, ICTAN-CSIC, 28040 Madrid, Spain; mmesias@ictan.csic.es; 4 Departamento de Nutrición y Bromatología, Instituto de Investigación Biosanitaria ibs.GRANADA, Universidad de Granada, 18071 Granada, Spain; jarufian@ugr.es

**Keywords:** Amadori compounds, hydroxymethylfurfural, bread crust, Maillard reaction products (MRP), Caco-2 cells, availability, reactive oxygen species (ROS), protective action

## Abstract

Bread crust is one of the major contributors to the intake of Maillard reaction products (MRP). MRP improve the organoleptic properties of foods and can provide biological actions such as antioxidant properties. The transport and availability of Amadori compounds (measured as furosine) and hydroxymethylfurfural (HMF)—early and intermediary MRP—from enzymatically digested bread crust (BC) and from its soluble low-molecular weight (LMW) and high-molecular weight (HMW) fractions were investigated in the Caco-2 cell line. The absorption of the early and final MRP pool was tested by measuring the absorbance recovery (280 and 420 nm). The ability of soluble BC or its fractions to lessen the production of reactive oxygen species (ROS) was examined. Amadori compounds (furosine) were transported across Caco-2 cell monolayers from the soluble BC in percentages ranging between 40% and 56%; the lower amount of the compound supplied, the higher transport rate. However, HMF transport rate (35%) was unaffected by the initial amount of the compound. Amadori compounds and HMF contained in the LMW fraction were more efficiently transported than those present in the HMW fraction, suggesting improved absorption when supplied as free forms or linked to LMW compounds. Absorbance recovery at 280 nm was higher from the LMW fraction, whereas higher recovery was detected for the HMW fraction at 420 nm. The digested BC—but not its isolated fractions—was able to significantly reduce ROS production at basal conditions and after subjecting cells to an oxidant. A clear positive action of BC on the antioxidant defence is manifested, seemingly attributable to the combined presence of soluble LMW and HMW products.

## 1. Introduction

Bread baking induces many different modifications in the chemical composition and properties of the original food matrix [1,2], and definitively changes the nutritional value of the final product. It is well-known that caramelisation and the Maillard reaction (MR) are directly involved in these changes, producing newly formed compounds responsible for different biological activities [3] and improving the organoleptic characteristics of bread, such as its appearance and taste [4,5]. Antioxidant capacity is within those interesting biological activities associated with neo-formed compounds [4,6,7], although other bread components such as phenolic compounds [8], fibre [9], or tocols [10] can also contribute to this capacity. The outstanding feature of Maillard reaction products (MRP) is their generation during heat treatment, while, on the contrary, many of the former are degraded by heating [11]. Insofar as these neo-formed products are promoted by heating, their abundance is higher in the bread crust, since this is the surface with greater exposure to heat during baking. Amadori compounds must be mentioned between the earlier products formed exclusively via MR, while hydroxymethylfurfural (HMF)—coming from more advanced stages—is generated from both the MR and caramelisation. The appearance of the first is an important nutritional aspect, because a decrease in the availability of participating amino acids occurs [12]. On the other hand, their intake and absorption deserves attention, since they can act as seeds for the in vivo formation of some other products, known as advanced glycation end-products (AGEs) (e.g., carboxymethyl-lysine, considered as markers for ageing and often associated with age-related degenerative diseases) [13]. However, some positive actions have also been linked with AGEs. A recent study developed in mice and cardiac fibroblasts derived from them have established that RAGEs (the best-characterized AGE-receptor) are activated by dietary bread crust-derived AGEs. Next, the induction of antioxidant defence through the activation of the gene expression of superoxide dismutase takes place, suggesting that bread crust might be useful as a preconditioning agent to protect cardiac cells against ischemia–reperfusion damage [14].

Regarding Amadori products, they are analytically determined as furosine, the artificial amino acid formed during acid hydrolysis of the Amadori compounds, originated in the interaction of the ε-amino groups of lysine with glucose, lactose, and maltose [15]. Furosine has been considered as a useful indicator of the degree of damage during the initial steps of the MR in cereal products such as bread [16] and breakfast cereals [17]. Although less valuable as an index of thermal damage in cereal-derived products, HMF is increasingly important due to the possible mutagenic activity of its in vivo metabolites [18,19]. The mechanisms of the toxic activity of HMF remain unclear: It has been proposed that it is metabolically activated through the sulfonation of its allylic hydroxyl functional group, which can take place in the liver by hepatic sulfotransferase. The electrophilic intermediate obtained—5-sulfooxymethylfurfural (SMF)—can interact with critical cellular nucleophiles (e.g., DNA, RNA, and proteins), resulting in structural damage, which can lead to toxicity and mutagenicity [20]. 

Due to the complexity of designing and developing proper human trials, limited data are available in the scientific literature on the bioavailability of MRP contained in foods or meals (e.g., [21]). Lacking that, experiments in cell cultures are given as a valid approach to meet this challenge. Particularly, the Caco-2 cell line—which exhibits in culture many properties of normal intestinal epithelium—has been widely used as a suitable model to study nutrient and non-nutrient absorption and metabolism [22,23]. A recent study on HMF availability from digested breakfast cereals using the Caco-2 cell line has established a low HMF availability from these foods, highly influenced by the composition [24]. The mentioned cell model is also suitable for the investigation of the oxidative stress caused by different compounds, MRP among them [25].

The goal of the present work was to examine Amadori products (measured as furosine) and HMF availability from in vitro digested bread crust in biological systems using a human intestinal cell culture model, the Caco-2 cell line. In order to analyse the global transport of polled early and final MRP across cell monolayers, the absorbance recovery was tested at 280 and 420 nm in the cell assay. The effects of digested bread crust on the generation of reactive oxygen species (ROS) were also analysed in this model. Special attention was paid to the high and low molecular weight compounds present in the bioaccessible fraction of bread crust, which were isolated and studied separately to identify factors responsible for the possible effects.

## 2. Materials and Methods

### 2.1. Chemicals

All chemicals for reverse phase-high performance liquid chromatography (RP-HPLC) determinations were from Merck (Darmstadt, Germany) unless mentioned otherwise. Ethanol, methanol, 2,2-diphenyl-1-picrylhydrazyl (DPPH), and 2,4,6-tri(2-pyridyl)-s-triazine (TPTZ) were purchased from Fluka Chemicals (Madrid, Spain). The standard 6-hydroxy-2,5,7,8-tetra-methyl-chroman-2-carboxylic acid (Trolox) and all cell culture media, cell culture-grade chemicals, and those for in vitro digestion were obtained from Sigma Chemical Co. (St. Louis, MO, USA). Culture flasks were purchased from Corning Costar (Cambridge, MA, USA). 

### 2.2. Extraction of Bread Crust and Its Soluble Fractions with Different Molecular Weights

Bread crust (BC) was supplied by a Spanish manufacturer of cereal-derived food products. The cleaning of the bread crust from the attached bread crumb as well as the production of its different soluble high and low molecular weight fractions are largely described in Roncero-Ramos et al. [26]. Briefly, in order to separate the MRP, a portion of bread crust was enzymatically hydrolysed with pronase E (4,000,000 units/g), applying an appropriate concentration and incubation time [27]. The soluble fraction (42.48% *w*/*w* of bread crust) was subjected to ultrafiltration, as described in the above-mentioned study. The fraction constituted by compounds with a molecular mass higher than 5 kDa was retained (called high molecular weight, HMW, 57.14% *w*/*w* of the bread crust) and the fraction containing compounds with a mass of less than 5 kDa was filtered (called low molecular weight, LMW, 42.86% *w*/*w* of the bread crust). Both fractions, together with the cleaned BC, were lyophilized, powdered, and homogenized, and stored at −20 °C until used in our assays.

### 2.3. Characterization of Bread Crust and the LMW and HMW Fractions

Nitrogen and carbon were analysed in BC, HMW ,and LMW samples in a LECO model FP-2000 protein/nitrogen analyser (Leco Instruments, Madrid, Spain) calibrated with ethylendiaminetetraacetic acid (EDTA) (Dumas method).

In order to extract antioxidants from the samples (BC, HMW, and LMW), a chemical extraction was performed following the procedure described by Pérez-Jiménez and Saura-Calixto [28]. Briefly, 0.5 g of fresh sample was placed in a tube, and 5 mL of acidic methanol/water (50:50 *v*/*v*, pH 2) were added. The tube was thoroughly shaken at room temperature for 1 h and centrifuged at 2500× *g* for 10 min, and the supernatant was recovered. Five millilitres of acetone/water (70:30, *v*/*v*) were added to the residue, and the shaking and centrifugation steps were repeated. The methanolic and aqueous-acetone extracts were then combined, and the volume made up to 10 mL. The antioxidant capacity assays on these samples were performed as stated previously [29]. Concisely, the DPPH method was carried out by adding a 500 µL aliquot of sample or Trolox standard to 1 mL of DPPH (74 mg/L in methanol). A daily-prepared solution of DPPH gave a final absorption at 520 nm of 1.8 AU. Mixture was shaken for 1 h, and then absorption was measured at 520 nm. Temperature in the measurement chamber was set at 30 °C. Aqueous solutions of Trolox at various concentrations were used for calibration (0.15–1.15 mM). The results were expressed as mmol equivalents of Trolox per kg of sample. In the case of the ferric reducing ability of plasma (FRAP) method, briefly, 900 µL of FRAP reagent—freshly prepared and warmed at 37 °C—was mixed with 150 µL of test sample, Trolox standard, or water as appropriate reagent blank. The FRAP reagent contained 2.5 mL of a 10 mM TPTZ solution in 40 mM HCl plus 2.5 mL of 20 mM FeCl_3_∙H_2_O and 25 mL of 0.3 M acetate buffer, pH 3.6. Readings at the absorption maximum (595 nm) were taken every 15 s using a Perkin–Elmer Lambda 25 UV–Visible spectrophotometer (Perkin-Elmer, Waltham, MA, USA) equipped with a thermostated automatic sample positioner. Temperature was maintained at 37 °C, and the reaction was monitored up to 30 min. Trolox stock solutions were again used to perform the calibration curves. Results were also expressed as mmol equivalents of Trolox per kg of sample.

To evaluate the presence of MRP, Amadori compounds (as furosine) and HMF were analysed in the three samples (BC, HMW, and LMW). Furosine determination was performed following the method described by Resmini and Pellegrino [30] in order to avoid interference with other furoylmethyl-aminoacids, which could interfere in the faster capillary electrophoresis method [31]. Samples containing 6.5 mg of protein per mL of 7.95 M HCl were bubbled with high purity N_2_ gas for 2 min and then hydrolysed at 120 °C for 23 h in a Pyrex screw-cap vial with polytetrafluoroethylene (PTFE)-faced septa. The hydrolysate was filtered with a medium-grade paper filter. A 0.5 mL portion of the filtrate was applied to a Sep-pack C_18_ cartridge (Millipore, Danvers, MA, USA) prewetted with 5 mL of methanol and 10 mL of deionized water, and was then eluted with 3 mL of 3 M HCl. Then, the acidic eluate was dried in a rotary evaporator at 40 °C and re-dissolved in 100 µL of acetonitrile:water (20:80 *v*/*v*) mixture. Fifty microliters of the solution were analysed by ion-pair RP-HPLC, which consisted of an Accela 600 system (Thermo-Scientific, Waltham, MA, USA) with a diode-array detector, Waters model 600 quaternary gradient bomb (Waters, Milford, MA, USA), and a UV/VIS detector model 200 from Konik (Barcelona, Spain) set at 280 nm. The analytical column was a C_18_ analytical column (Novapack C_18_, 250 × 4.6 mm, 4 µm particle size, Waters, Milford, MA, USA) thermostated at 32 °C. The UV detector was set at 280 nm, and 25 µL were injected. Furosine quantitation was performed by the external standard method within the range 0.01–1000 mg/L, using a commercial standard of pure furosine (Neosystem Laboratoires, Strasbourg, France). The analysis was performed in duplicate, and the data are the mean values expressed as milligrams per 100 g of protein. The determination of the second marker, HMF, was based on the method of Rufián-Henares et al. [32] with slight modifications [33]. Ground sample (1.0 g) was suspended in 7 mL of deionized water in a 10 mL centrifuge tube, and the tube was shaken vigorously for 1 min. The resulting mixture was centrifuged at 4500× *g* for 10 min at 4 °C. The supernatant was collected on a 10 mL volumetric flask, and another two extractions were performed by adding 2 mL of deionized water. The supernatants were mixed and clarified with 0.250 mL of each Carrez I (potassium ferrocyanide, 15% *w*/*v*) and Carrez II (zinc acetate 30% *w*/*v*) solutions. After a final centrifugation, the volume was completed up to 10 mL with deionized water. Then, 2 mL were filtered (0.45 µm) to analyse the HMF content. The HPLC system was similar to that used for furosine analysis. The mobile phase was made up of a mixture of acetonitrile in water (5% *v*/*v*) delivered at the flow rate of 1 mL/min under isocratic conditions through a C_18_ analytical column (Novapack C_18_, 250 × 4.6 mm, 4 µm particle size, Waters, Milford, MA, USA) thermostated at 32 °C. The UV detector was set at 284 nm, and 20 µL were injected. The analysis was performed in duplicate, and HMF was quantified by the external standard method within the range 0.01–50.00 mg/L.

### 2.4. Bioaccessibility of Amadori Compounds and HMF from Bread Crust 

After the enzymatic digestion of BC with pronase E as described previously, the soluble fraction was separated to analyse the soluble portion of Amadori compounds (measured as furosine) and HMF. Since the measurement was performed in the supernatant obtained after digestion, the preparation of samples was slightly different from that mentioned above. In the case of furosine determination, 0.3 mL of sample were hydrolysed with 1 mL of 7.95 M HCl, and finally 25 µL of the HCl free solution obtained after rotary evaporation were analysed by ion-pair RP-HPLC with the same analytical system. Furosine quantitation was performed as described above, but building a specific calibration curve within the range 0.0001–1.0000 mg/L. For the HMF analysis, 0.3 mL of supernatant were clarified with 0.050 mL each of Carrez I and II solutions. The resulting mixture was centrifuged at 4500× *g* for 10 min at 4 °C. Two hundred microliters of the solution were filtered (0.45 µm) to analyse the HMF content in the same HPLC system. HMF was again quantified by the external standard method within the range 0.0001–1.0000 mg/L.

Amadori compounds (as furosine) and HMF analyses were performed in duplicate, and the data of their bioaccessible fractions from BC are the mean values expressed as milligrams per kg of BC. 

### 2.5. Cells

Cell culture. Caco-2 cells were purchased from the European Collection of Cell Cultures (ECACC) through the Cell Bank of Granada University, at passage 20, and used in experiments at passages 22–30. Cells were grown in 75-cm^2^ plastic flasks containing high-glucose Dulbecco’s modified minimal essential medium (DMEM), with heat-inactivated fetal bovine serum (15%), NaHCO_3_ (3.7 g/L), nonessential amino acids (1%), 4-(2-hydroxyethyl)-1-piperazineethanesulfonic acid (HEPES) (15 mM), bovine insulin (0.1 UI∙mL/L), and 1% antibiotic–antimycotic solution. The cells were maintained at 37 °C in an incubator with an atmosphere of air/CO_2_ (95:5) at 90% humidity, and the medium was changed every 2 days. 

Trypsinization and seeding of cells were performed as described elsewhere [34]. At 80% confluency, cells were collected and seeded into bicameral chambers (Transwell, 24-mm diameter, 4.7-cm^2^ area, 3-μm pore size, Costar) at a density of 75,000 cells/cm^2^, with 2.5 mL of medium in the well (basolateral cell side) and 1.5 mL of medium in the insert (apical cell side). The medium was changed every 2 days and the day before cultures were used for transport experiments. The development of functional tight junctions and cell monolayer integrity during differentiation of Caco-2 cells was monitored by measuring absorption of the phenol red marker, as described previously [35]. Cell monolayers were used for absorption study when the leakage rate of phenol red was lower than 2.5% per hour.

### 2.6. Amadori Compounds and HMF Absorption Experiments

Experiments were carried out twenty-one days after initial seeding. Spent culture medium was aspirated from the apical and basolateral chambers, and both cell surfaces of the monolayer were washed two times with Hank’s balanced salt solution (HBSS) at 37 °C. The transport solution (2.5 mL) was added to the basolateral chamber, and the test transport solutions were added to the apical chamber (1.5 mL). The test transport solutions were as follows: (i) the supernatants of the enzymatically digested BC diluted 1:3 and 1:9 (*v*/*v*) with dilution solution (130 mM NaCl, 5 mM glucose, and 50 mM HEPES, pH 7) to facilitate cell viability (termed as soluble bread crust (SBC)-A and SBC-B, respectively); (ii) the isolate LMW soluble fraction of BC dissolved in the dilution solution in the same amount (mg of powder fraction) as it was present in SBC-B (termed as LMW-B), calculated taking into account the presence of this fraction in the soluble fraction of bread crust; (iii) the isolate HMW soluble fraction of BC dissolved in the dilution solution in the same amount (mg of powder fraction) as it was present in SBC-B (termed as HMW-B), calculated as mentioned previously; (iv) additionally, the isolate HMW soluble fraction of BC dissolved in the dilution solution in the same amount (mg of powder fraction) as that used for the preparation of the LMW-B solution (termed as HMW-C), aiming to understand the effects of the isolate fractions when supplied to cells in equal amount.

Cell cultures were then incubated at 37 °C in humidified air: CO_2_ atmosphere for 2 h. Cell viability assays by trypan blue exclusion were carried out after 2 h of exposure to the test transport solutions, and it was never <85%. After incubation, medium from the apical compartment was aspirated. To calculate the Amadori compounds (measured as furosine) and HMF transported across the cell monolayer, the transport solution from the basolateral chamber was removed. 

Amadori compounds (as furosine) and HMF were measured in the diluted digests, in the initial LMW and HMW solutions, and in the transport solutions removed from the basolateral chamber by RP-HPLC using the protocol described for liquid samples. 

In order to non-specifically check the transport of pooled early and final MRP (especially melanoidins) with maximum absorbance at 280 and 420 nm, respectively, absorbance measurement at both wavelengths were carried out in solutions before the experiments and in the transport buffer removed from the basal chamber after the incubation time. The absorbance recovery (%) was calculated taking into account the corresponding dilution factors.

### 2.7. Reactive Oxygen Species Generation

ROS generation was determined by the dichlorofluorescein (DCFH) assay described by Goya et al. [36] with modifications. The procedure is based on the use of the chemical 2′,7′-dichlorofluorescin diacetate (DCFH-DA; Sigma Chemical Co., St. Louis, MO, USA)—a cell-permeable non-fluorescent probe which is de-esterified intracellularly and turns to highly fluorescent 2′,7′-dichlorofluorescein (DCFH) upon oxidation. Cells were seeded in 24-well multiwell plates at a density of 2 × 10^5^ cells per well in 1 mL of the medium, and incubated at 37 °C for 48 h. The cells were pretreated with 1 mL of the soluble fraction of BC after enzymatic digestion (SBC-A or SBC-B) or the soluble LMW and HMW compounds isolated from BC in different proportions, as described previously (LMW-B, HMW-B, or HMW-C) and incubated for 2 h. The control wells received fetal bovine serum-free DMEM. The medium was then discarded, and the cells were treated with DFCH-DA 10 μM and incubated for 1 h. The DCFH-DA was removed, and culture medium (for basal measurements) or the oxidizing agent hydrogen peroxide (H_2_O_2_) 5 mM (to study the protective effect against oxidation) were added to the wells. The fluorescence was immediately measured in the plate reader at a wavelength of 485 nm excitation and 535 nm emission, at a constant temperature of 37 °C for 90 min. DCFH-DA is converted into dichlorofluorescein (DCFH) in the presence of ROS, and emits fluorescence.

### 2.8. Statistical Treatment

Statistical significance of data was tested by one-way analysis of variance (ANOVA) followed by the Duncan Test to compare means that showed significant variation (*p* < 0.05). When the comparisons were carried out exclusively between SBC-A and SBC-B, a Student’s *t*-test was applied. Analyses were performed using Statgraphics Plus, version 5.1, 2001 (Statpoint Technologies, Warrenton, VA, USA).

## 3. Results and Discussion

### 3.1. Sample Characterization and Bioaccessibility of Amadori Compounds and HMF from Bread Crust

Before conducting the cell assays, the characterization of the initial samples was performed. Table 1 depicts data obtained for BC before and after digestion, termed in the table as “bioaccessible fraction”, as well as for LMW and HMW fractions. Nitrogen content was especially elevated in the LMW sample. This fact suggests that pronase E digestion led to a significant amount of soluble nitrogen likely corresponding to amino acids being released after the enzymatic action. This could be explained by the fact that pronase has a broad specificity, breaking down virtually all proteins into their individual amino acids. Carbon was higher in the complete BC, probably because large structures combining protein and carbohydrate were present before digestion; e.g., melanoproteins, a proteinaceous material representing the main melanoidin fraction of bread [37]. After the enzymatic hydrolysis, a higher amount of carbon was detected in the soluble HMW fraction, a fact linked to the presence of fibre in bread within which melanoidins are sometimes considered. Studies on bread and coffee melanoidins suggest that these Maillard polymers resemble the dietary fibre present in heated products [38].

Most natural antioxidants are multifunctional, and in complex heterogeneous foods, their activity cannot be evaluated by a unique method. In the present study, two different methods were used to measure the antioxidant activity of BC, LMW, and HMW samples: the free radical scavenging capacity was tested by the DPPH method, whereas the iron reducing ability was measured by the FRAP protocol. The undigested BC exhibited the higher free radical scavenging ability (*p* < 0.05), followed by the soluble LMW fraction. The soluble HMW fraction presented scant scavenging capacity. However, regarding the ability to reduce iron, the BC sample showed the lowest power (*p* < 0.05), while its isolated soluble fractions reported high values, especially in the case of the LMW sample. Previous works of our research group reported noticeably minor DPPH and FRAP values for commercialized pre-baked wheat bread finished in a home oven set at 200 °C for 26 min [39]. The discordance could be due to the consideration of isolate BC in the present study, in which neo-formed compounds with important antioxidant contribution are present in higher concentrations than when considering the whole bread, because of a dilution effect.

The amount of furosine (representing the Amadori products) and HMF in the initial samples are also depicted in Table 1. As a consequence of the enzymatic digestion of the proteins of BC and the subsequent isolation of each soluble fraction, values for LMW and HMW increased by three to four times those measured in the complete BC. The furosine level in BC was close to the variation described by Ramírez-Jiménez et al. [16] (75–221 mg/100 g protein), and was higher than the data reported by Hidalgo and Brandolini (170 mg/100 g protein) [40]. Likewise, Michalska et al. [4] remarked that rye breads had furosine levels ranging between 94.2 and 336.2 mg/100 g protein in the crust. According to these authors, variations between different breads can be attributed to the different reducing sugars and protein contents of the flours used. In the case of HMF, the level detected in this study for BC was in the range determined by Ramírez-Jiménez et al. [13] (18.3–176.1 mg/kg dry matter (DM)) and also to that stated by Hidalgo and Brandolini [40].

Once BC was submitted to the enzymatic digestion to obtain its soluble fraction, the level of bioaccessible Amadori products (as furosine) and HMF was calculated. Thus, only the 6.4% of furosine and the 4.0% of HMF present in BC were accessible to be absorbed. That soluble fraction properly diluted to ensure cell viability was subsequently used in the cell assays.

### 3.2. Amadori Compounds and HMF Transport in Cell Culture

Results of the study of MRP transport in the Caco-2 cell line are included in Table 2. In order to assay the effects of concentration, two different viable dilutions of the soluble fraction of BC were tested in the transport assays: 1:3 and 1:9 (*v*/*v*) (SBC-A and SBC-B solutions). As a consequence of the dilution, the amount of Amadori compounds (as furosine) and HMF offered to the cells was significantly different (*p* < 0.05). It was observed that the transport of Amadori compounds was significantly higher when the dilution factor increased (40% vs. 56% transport rate for SBC-A and SBC-B, respectively), perhaps pointing to some kind of competitive mechanism for its transport which could be saturated. Thus, lower amounts of Amadori products would be more efficiently absorbed than higher ones. Studies by Seidowski et al. [41] have recently established that complex Amadori products linked to oligosaccharides can be slowly but efficiently hydrolysed by α-glucosidase activity (sucrase-isomaltase) expressed by Caco-2 cells, a more efficient conversion when a lower amount of substrate is supplied to the cell. In this way, released Amadori products and monosaccharides are more easily absorbed during their intestinal passage. Unlike the modifications found in the Amadori compounds’ transepithelial flux, the transport rate of HMF was unchanged when SBC-A or SBC-B solutions were administered, and the data were very close to the transport rate obtained by our research group in previous Caco-2 cell assays using in vitro gastrointestinally digested breakfast cereals (23%–32% of HMF transport, depending on the composition) [21]. Similarly, other researchers have established transport rates of around 20% for this compound when a 100 µM HMF solution was supplied to the cells during 4 h of incubation time [42]. They also worked with other fragrant 3(2*H*)-furanones (e.g., 4-hydroxy-2,5-dimethyl-3(2*H*)-furanone, HDMF) at different concentrations (100 and 500 μM) aiming to test whether the transport of 3(2*H*)-furanones could be saturated by high levels, but saturation of the transport was not observed [42]. On the basis of this statement, our unmodified HMF transport rate (35%) for the SBC-A solution and its dilution SBC-B could be understood. Regarding the absorption mechanism, Stadler et al. [42] described that since transport could not be saturated by high levels of the substances, and because they observed bidirectional transport to the apical chamber, a directed active transport is unlikely. Therefore, passive diffusion by paracellular transport is proposed. 

**Figure 1:**
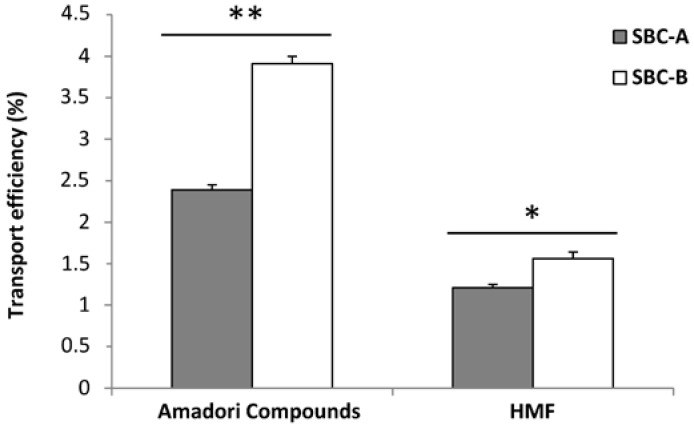
Transport efficiency (%) of Amadori Compounds (measured as furosine, the artificial amino acid formed during acid hydrolysis of this product) and HMF after 2 h exposure to soluble fraction of bread crust at two different concentrations. Values are mean ± standard error (SE) (*n* = 3). The asterisk on the bars of a compound indicates significant differences between samples (* = *p* < 0.05; ** = *p* < 0.001). SBC-A: soluble bread crust fraction dilution 1:3 (*v*/*v*); SBC-B: soluble bread crust fraction dilution 1:9 (*v*/*v*).

Taking into account the Amadori products (as furosine) and HMF solubility after the enzymatic digestion and their transport rate in the cell assay, the transport efficiency could be calculated (Figure 1), expressed as the percentage of Amadori compounds or HMF transported from the total present in the BC. Since two different dilutions of the soluble fraction of BC were used in the transport experiments, two values were obtained for the transport efficiency of Amadori compounds (as furosine) and HMF coming from the SBC-A and SBC-B solutions, although figures are very close between them. In both cases, the transport efficiency was higher (*p* < 0.05) from the SBC-B solution, indicating that the greater dilution factor increased the availability of the compounds. When comparing Amadori products vs. HMF transport efficiency, the former was significantly higher (*p* < 0.05). Although data are quantitatively low, the results must be interpreted due to the small surface of the wells used for the assay (4.7 cm^2^), and that cells were seeded at a density of 75,000 cell/cm^2^. It is considered that the intestinal surface area (including microvilli) is about 300 m^2^ [43]; therefore, it could be supposed that in vivo the results would be modulated by that higher surface area. In the same range of data found in the present work, Delgado-Andrade et al. [24] established HMF transport efficiencies between 8.42% and 2.06% for different breakfast cereals.

To determine if a matrix effect could affect the Amadori products and HMF transport, isolated soluble LMW and HMW fractions of BC (LMW-B and HMW-B solutions) were used in the transport experiments. It is important to remark that the amounts of LMW and HMW samples present in them were both proportional to that found in combined form in SBC-B. Based on this premise, the initial amount of products supplied to cells was different and led to different quantities of Amadori products (as furosine) and HMF transported (*p* < 0.05) (Table 2). When expressed as a percentage of the initial furosine and HMF set at the apical chamber, it could be observed that the transport rate doubled in the case of the LMW-B solution compared with the HMW-B one for both compounds. The more plausible hypothesis to understand this result would be that free and/or simple forms of Amadori products and HMF would be present in the soluble LMW fraction of BC, and then their absorption would be enhanced. 

To complete the experimental design, an additional solution was prepared from the HMW powder sample, the HMW-C solution. Amadori compounds and HMF transport could be affected by the form in which they are present in each fraction (hypothetically in free or simple forms in the case of the LMW fraction or protein-bound or other complex forms in the case of the HMW fraction) and by the amount of these fractions supplied to the cells, which could modify the absorption rate by competition mechanisms. Because of that, the HMW-C solution—containing the same amount (milligrams) of HMW sample as that used from the LMW sample to prepare the LMW-B solution—was supplied to the cells. It was observed that the transport rate of Amadori compounds (as furosine) and HMF was significantly lower (*p* < 0.05) from HMW-C than from LMW-B solution, confirming the importance of the chemical form of the compounds in their absorption. Moreover, the transport rate was not significantly different between HMW-B and HMW-C solution (with significantly different initial Amadori compounds and HMF amounts), supporting the statement of Stadler et al. [42] regarding the unsaturation of the HMF transport.

Concerning the absorbance recovery in the basal chambers (Table 3), a significant increase (*p* < 0.05) was shown in the SBC-B solution compared with the SBC-A one at 280 nm (1.6% vs. 10.2%)—a result probably linked to the dilution factor. The LMW-B solution depicted the highest recovery at 280 nm, suggesting that soluble early and intermediate Maillard compounds with lower molecular weights were more easily absorbed than soluble HMW products. Regardless of the initial absorbance detected in the apical chamber, HMW-B and HMW-C solutions exhibited the same absorbance recovery at 280 nm. As a general conclusion, results of absorbance recovery at 280 nm followed the same pattern of that observed for furosine and HMF in the transport experiment, a logical fact considering that these compounds were measured by chromatographic procedures using that wavelength. Curiously, when the recovery of final browning compounds was measured at 420 nm, HMW solutions presented the highest recoveries, even though these compounds would have greater complexity than those present in the LMW-B solution. At the mentioned wavelength, the dilution factor did not influence the transport of browning products between SBC-A and SBC-B solutions; however, it did indeed in the case of HMW-B vs. HMW-C solutions, improving the absorbance recovery in the latter (*p* < 0.05).

### 3.3. ROS Generation

ROS production can induce oxidative stress, leading to cell damage that can culminate in cell death. This damage is linked to the onset of many degenerative diseases, including cancer, cardiovascular disease, cataracts, and aging. Antioxidants can attenuate the damaging effects of ROS and delay many events that contribute to cellular aging [44]. Under basal conditions, the 2 h incubation of cells with SBC-A and SBC-B solutions led to a significant reduction in ROS, with a larger decrease in the former (Table 4). However, if soluble fractions of BC were supplied separately to the cells, the ROS production reached the same values as those measured in the Control. When comparing these data with the results found after the DPPH assay in the samples (Table 1)—a method testing the free radical scavenging capacity—a similar trend in the initial samples was observed. In order to induce oxidative stress, the differentiated Caco-2 cells were treated with 5 mM H_2_O_2_ for 2 h. The damage produced by the oxidant provoked an increase in ROS generation in the cells, and thus enabled us to estimate the protective effect of test solutions. Again, the same pattern described previously in the basal assay was observed. SBC-A and SBC-B solutions were more efficient in the reduction of the ROS generation, with a higher decrease in the case of SBC-A (*p* < 0.05), the most concentrated solution and therefore with higher presence of potential antioxidant compounds. These findings confirm the positive action of soluble BC at the cellular level, but not its isolated fractions, suggesting some kind of beneficial effect derived from the combined presence of LMW and HMW in the soluble BC, which is not found in each of them in a separate form.

In this context, Lindenmeier et al. [45] reported that the process of baking bread produced a novel type of antioxidant—the Maillard-derived product pronyl-lysine—which was eight times more abundant in the crust than in the crumb. Using human intestinal cells, this compound was shown to be the most effective component in bread for boosting the level of phase II enzymes, which has a well-known role in cancer prevention. The effects of BC in antioxidant defence have been recently studied by Pötzsch et al. [46], concluding that the presence of carboxymethyllysine-modified gliadin (another Maillard derivative) is also responsible for the antioxidative capacity and for the induction of the NF-kB pathway in mouse macrophages. 

Concerning animal trials, a previous study of our scientific team established that the ingestion of MRP derived from BC by rats induced a clear increase in the catalase and glutathione peroxidase activities, as well as higher levels of reducing power (glutathione, GSH) within the hepatic cell, revealing a positive action of those specific compounds at this level [47]. On the other hand, the consumption of the glycose-lysine MRP in the diet did not affect the hepatic antioxidant defence of Wistar rats, while some positive modifications (e.g., an increase in glutathione peroxidase) were detected in muscle (29%) and serum (400%), pointing to an improved antioxidant capacity after the consumption of those model MRP [48]. On the other hand, as previously mentioned, the work by Leuner et al. [14] in mice and their cardiac fibroblasts demonstrated that bread crust feeding induced the activation of the antioxidant defence by the increase in the gene expression of superoxide dismutase. The authors suggested that bread crust might be a promising tool as a preconditioning agent to protect cardiac cells against ischemia–reperfusion damage.

## 4. Conclusions

The present study shows that BC is a source of absorbable Maillard compounds in the diet, as evidenced by our transport assays performed in the Caco-2 cells line in the case of Amadori products and HMF, as well as in the absorbance recovery trials at 280 nm (early compounds) and 420 nm (final products). Although the results suggest low transport efficiencies, data must be interpreted considering the context of the real intestinal surface (around 300 m^2^ including microvilli), which could definitively modulate their absorption. Moreover, likely due to the participation of saturable transport mechanisms, a negative effect of the concentration was detected. A clear positive action of soluble BC on the antioxidant defence of this cell line is manifested. This fact cannot be exclusively linked to a specific fraction of compounds, but a combined action of soluble LMW and HMW products present in BC. 

## Figures and Tables

**Table 1:** Sample characterization.

Samples	N (%)	C (%)	DPPH (mmol·Trolox/kg)	FRAP (mmol·Trolox/kg Powder Sample)	Amadori Compounds (as Furosine) (mg/100 g Protein in Powder Sample)	Amadori Compounds (as Furosine ) in Bioaccessible Fraction (mg/kg Powder BC)	Bioaccessible Amadori Compounds (as Furosine) (%)	HMF (mg/kg Powder Sample)	HMF in Bioaccessible Fraction (mg/kg Powder BC)	Bioaccessible HMF (%)
BC	2.32 ± 0.03 ^b^	44.60 ± 0.01 ^c^	10.88 ± 0.34 ^c^	98.5 ± 3.4 ^a^	377.9 ± 12.3 ^a^	31.7 ± 3.9	6.41 ± 0.52	27 ± 0.3 ^b^	1.1 ± 0.2	4.03 ± 0.46
LMW	4.36 ± 0.04 ^c^	35.95 ± 0.07 ^a^	9.01 ± 0.42 ^b^	206.5 ± 6.8 ^c^	906.0 ± 41.7 ^b^	-	-	40 ± 0.6 ^c^	-	-
HMW	2.20 ± 0.01 ^a^	38.95 ± 0.07 ^b^	0.92 ± 0.01 ^a^	167.7 ± 4.3 ^b^	1376.1 ± 38.2 ^c^	-	-	20 ± 0.3 ^a^	-	-

BC: Bread crust, which was submitted to enzymatic digestion to obtain its soluble fraction; HMW: soluble high molecular weight fraction isolated from BC; LMW: soluble low molecular weight fraction isolated from BC; N: Nitrogen; C: Carbon; DPPH: 2,2-diphenyl-1-picrylhydrazyl; FRAP: Ferric reducing ability of plasma; Furosine is the artificial amino acid formed during acid hydrolysis of the Amadori compounds; HMF: hydroxymethylfurfural. Values are mean ± SD (*n* = 3). Different letters in each column indicate significant differences between samples (*p* < 0.05). Hyphen indicates the parameter has no interest in that sample.

**Table 2:** Amadori compounds (as furosine ^1^) and HMF transport in Caco-2 cell monolayer after 2 h of incubation with digested bread crust or its fractions.

Solutions	Amadori Compounds Transport (as Furosine ^1^)		HMF Transport	
	ng/well	%	ng/well	%
SBC-A	303 ± 8 ^a^	40.1 ± 1.1 ^a^	8.6 ± 0.3 ^a^	35.5 ± 1.2 ^d^
SBC-B	211 ± 4 ^b^	56.0 ± 1.2 ^b^	4.8 ± 0.2 ^b^	35.4 ± 1.6 ^d^
LMW-B	118 ± 1 ^c^	99.2 ± 1.2 ^c^	3.2 ± 0.2 ^c^	96.0 ± 6.7 ^a^
HMW-B	109 ± 3 ^c^	55.6 ± 1.5 ^b^	1.1 ± 0.1 ^d^	55.6 ± 4.3 ^c^
HMW-C	73 ± 1 ^d^	55.6 ± 1.1 ^b^	0.8 ± 0.0 ^d^	69.4 ± 6.9 ^b^

^1^ Furosine is the artificial amino acid formed during acid hydrolysis of the Amadori compounds. Values are mean ± SE (*n* = 3). Different letters in each column indicate significant differences between samples (*p* < 0.05). HMF: hydroxymethylfurfural; SBC-A: soluble bread crust fraction dilution 1:3 (*v*/*v*); SBC-B: soluble bread crust fraction dilution 1:9 (*v*/*v*); LMW-B: soluble LMW fraction naturally present in SBC-B; HMW-B: soluble HMW fraction naturally present in SBC-B. HMW-C: soluble HMW fraction in the same amount of LMW-B.

**Table 3:** Absorbance recovery across the Caco-2 cell monolayers after 2 h of incubation with digested bread crust or its fractions.

Solutions	Apical Chamber	Basolateral Chamber	
	Initial Abs (AU)	Final Abs (AU)	Recovery (%)
280 nm			
SBC-A	73.06 ± 0.10 ^a^	1.140 ± 0.029 ^a^	1.56 ± 0.04 ^d^
SBC-B	3.768 ± 0.02 ^b^	0.384 ± 0.010 ^b^	10.19 ± 0.26 ^c^
LMW-B	2.620 ± 0.01 ^c^	0.391 ± 0.005 ^b^	14.94 ± 0.21 ^a^
HMW-B	2.384 ± 0.01 ^d^	0.306 ± 0.007 ^c^	12.82 ± 0.28 ^b^
HMW-C	1.787 ± 0.01 ^e^	0.219 ± 0.013 ^d^	12.24 ± 0.74 ^b^
420 nm			
SBC-A	2.346 ± 0.01 ^a^	0.070 ± 0.005 ^a^	3.00 ± 0.20 ^d^
SBC-B	1.126 ± 0.01 ^b^	0.046 ± 0.006 ^b^	4.11 ± 0.57 ^d^
LMW-B	0.283 ± 0.01 ^c^	0.031 ± 0.001 ^c^	10.95 ± 0.20 ^c^
HMW-B	0.154 ± 0.01 ^d^	0.032 ± 0.004 ^c^	21.00 ± 2.50 ^b^
HMW-C	0.109 ± 0.01 ^e^	0.032 ± 0.001 ^c^	29.66 ± 1.22 ^a^

AU: arbitrary units. Values are mean ± SE (*n* = 3). Different letters in each column indicate significant differences between samples (*p* < 0.05). SBC-A: soluble bread crust fraction dilution 1:3 (*v*/*v*); SBC-B: soluble bread crust fraction dilution 1:9 (*v*/*v*); LMW-B: soluble LMW fraction naturally present in SBC-B; HMW-B: soluble HMW fraction naturally present in SBC-B. HMW-C: soluble HMW fraction in the same amount of LMW-B.

**Table 4:** Reactive oxygen species (ROS) generation in Caco-2 cells after 2 h incubation with the bioaccessible portion of bread crust (BC) or its different low and high MW fractions.

Samples	ROS (Fluorescent Units × 10^4^)	
	Basal Effect	Protective Effect
Control ^1^	18.13 ± 2.79 ^a^	36.25 ± 2.02 ^a^
SBC-A	2.86 ± 0.44 ^c^	10.61 ± 0.59 ^c^
SBC-B	7.82 ± 1.20 ^b^	18.35 ± 1.02 ^b^
LMW-B	18.04 ± 2.78 ^a^	35.57 ± 1.98 ^a^
HMW-B	19.91 ± 3.07 ^a^	30.99 ± 1.73 ^a^
HMW-C	14.21 ± 2.19 ^a^	29.60 ± 1.65 ^a^

^1^ In the case of the basal effect, the Control represents the basal ROS generation during the incubation with Dulbecco’s modified minimal essential medium (DMEM), while for the protective effect, the Control represents the ROS generation after incubation with DMEM followed by the oxidizing agent. Values are mean ± SE (*n* = 3). Different letters in each column indicate significant differences between samples (*p* < 0.05). SBC-A: soluble bread crust fraction dilution 1:3 (*v*/*v*); SBC-B: soluble bread crust fraction dilution 1:9 (*v*/*v*); LMW-B: soluble LMW fraction naturally present in SBC-B; HMW-B: soluble HMW fraction naturally present in SBC-B. HMW-C: soluble HMW fraction in the same amount of LMW-B.
